# Relationship between sleep quality and duration and the incidence rate of arthritis: A prospective cohort study

**DOI:** 10.1097/MD.0000000000039641

**Published:** 2024-09-13

**Authors:** HaiTing Wu, XinHua Yuan, QingSong Fu

**Affiliations:** a Department of Spine Surgery, Ningbo No. 2 Hospital, Ningbo, Zhejiang, China; b Department of Orthopedics, Ningbo No. 2 Hospital, Ningbo, Zhejiang, China.

**Keywords:** arthritis, older adult people, prospective cohort, sleep duration, sleep quality

## Abstract

Little is known about the relationship between sleep duration and quality and the risk of arthritis in the older adult population of China. The participants were a prospective cohort of adults aged 65 years and over who had not had arthritis at baseline and had participated in follow-up surveys from 2011 to 2018 in the Chinese Longitudinal Health and Longevity Survey (CLHLS). A Cox proportional-hazards regression model was established. The dose–response relationship between sleep duration and the incidence of arthritis was analyzed. The results of a Cox proportional-risk regression model revealed that sleep duration and quality were associated with the risk of arthritis in the participants. Specifically, compared with participants with good sleep quality, those with poor sleep quality had a 38% higher risk of developing arthritis (hazard ratio [HR] = 1.38, 95% confidence interval [CI] = 1.09–1.74), and compared with participants with a sleep duration of 5 to 10 hours, those with a sleep duration shorter than 5 hours had a higher risk of developing arthritis (HR = 1.56, 95% CI = 1.27–1.91). Moreover, the results of a restricted cubic spline Cox proportional-hazards regression model showed that there was an approximately non-linear dose–response relationship between sleep duration and the incidence rate of arthritis (*P* nonlinear < .0001). Participants with poor sleep quality and a sleep duration shorter than 5 hours had a higher risk of developing arthritis than those with better sleep quality and a longer sleep duration.

## 1. Introduction

Arthritis is a common chronic disease that deteriorates the structure and function of joints and thus causes persistent pain and limits mobility in patients, thereby severely affecting their quality of life. From 1990 to 2019, the global prevalence of arthritis increased, especially in older adult populations, and this increase is closely related to global population aging.^[1]^ There are multiple methods that can be used to alleviate the symptoms of arthritis, but it cannot be cured. In China, arthritis has become one of the main causes of disability and places a large economic burden on society and families. Therefore, early prevention and treatment of arthritis has become an important goal in public health. In view of this, it is crucial to determine all of the causes and risk factors of arthritis and identify those that can be eliminated or modified to reduce the incidence of arthritis.

Arthritis has various causes, and lifestyle and daily habits, especially sleep habits, are considered important factors that affect the incidence of arthritis.^[2]^ This is because sufficient sleep is necessary for maintaining joint health and reducing inflammatory reactions. In addition, sleep aids in the repair and recovery of the body, which supports the health and function of joints. As humans age, sleep problems gradually increase, and approximately 40% of older adult people experience sleep problems. Moreover, insufficient sleep and poor sleep quality are associated with worsening of arthritis symptoms.^[3]^ In addition, compared with a sleep duration of 7 to 8 hours, a shorter or longer sleep duration may increase the risk of arthritis, possibly due to an increase in inflammatory reactions and immune dysfunction.^[4]^

Prospective studies have explored the relationship between sleep duration or quality and the incidence rate of arthritis.^[3,5–7]^ Evidence suggests that sleep duration or sleep quality is associated with the risk of arthritis, but no study has examined the correlation between sleep (both quality and duration) and the risk of arthritis in the older adult population of China. Therefore, in this study, we analyzed nationally representative population survey data from the Chinese Longitudinal Health Longevity Survey (CLHLS) to determine the relationship between sleep quality and duration and the incidence rate of arthritis in older adult Chinese people.

## 2. Methods

### 2.1. Data sources

The CLHLS is a nationally representative, prospective, and continuous cohort study that comprises over 9765 participants aged 47 to 114 years. The baseline survey collected the participants’ sociodemographic characteristics, health status, and daily activities. The CLHLS has been approved by the Biomedical Ethics Committee of Peking University (IRB00001052-13074), and all of the participants or their legal representatives provided written informed consent during recruitment.

We refined the above-mentioned set of participants by excluding those aged under 65 at baseline (n = 86), those with missing data at the first follow-up (n = 791), those with missing sleep-quality data (n = 22) or sleep-duration data (n = 66), and those with arthritis or unknown arthritis status at baseline (n = 1600). In addition, we excluded participants with unknown arthritis status at follow-ups or abnormal or missing follow-up durations (n = 356). This afforded 6844 participants, whose data were analyzed to determine the relationship between sleep quality and duration and the incidence rate of arthritis. The entire process followed for including and excluding participants is shown in Supplementary Figure S1, Supplemental Digital Content, http://links.lww.com/MD/N591.

### 2.2. Sleep quality and duration

The baseline assessment of the participants’ sleep quality was conducted by asking them “What is the quality of your sleep?,” and based on their responses, they were divided into 2 groups: a good sleep quality (very good and good sleep quality) group and a poor sleep quality (very poor and poor sleep quality) group. The baseline assessment of the participants’ sleep duration was conducted by asking them “How long do you usually sleep?” Based on their responses and the literature, they were divided into 3 groups: a sleep-deprivation group (sleep duration of < 5 hours per day), an adequate sleep group (sleep duration of 5–9 hours per day), and an excessive sleep group (sleep duration of > 9 hours per day). In addition, we performed a multivariate analysis in which sleep duration was regarded as a continuous variable, which allowed us to calculate the hazard ratios (HRs) associated with sleep duration.

### 2.3. Outcome

The definition of an arthritis event was a participant’s report that they had been diagnosed with arthritis in a hospital. The arthritis status of deceased participants was obtained by asking their close relatives whether the participants had had arthritis during their lifetime. While we attempted to verify this information through community doctors when possible, it was not always supported by medical reports, which could introduce recall bias. Other information about deceased participants was obtained from family members or community doctors in subsequent interviews.^[8]^

### 2.4. Covariates

A questionnaire was used to collect information on individual-level covariates that may confound the relationship between sleep and arthritis, namely age (≤ 80 years or above), gender (female or male), place of residence (urban or rural), smoking status (current, previous, or never), alcohol-drinking status (yes or no), physical activity status (current, previous, or never), marital status, education level, body mass index (BMI), and household income. Marital status was classified as “married” (married and living with a spouse) or “other” (widowed/separated/divorced/never married/married but not living with a spouse). Education level was classified as no formal education (0 years), primary education (1 to 6 years), or higher education (6 years or more). Household income was classified as <4000 RMB (Renminbi), <10,000 RMB, <20,000 RMB, or equal to or >20,000 RMB.^[9]^ BMI was calculated as weight (kg)/height (m)^2^ and was classified as obese (≥28 kg/m^2^), overweight (24–<28 kg/m^2^), normal weight (18.5–<24 kg/m^2^), or underweight (<18.5 kg/m^2^). All BMI classifications aside from normal BMI were considered abnormal. In addition, history of heart disease (yes/no), diabetes (yes/no), and stroke (yes/no) were self-reported in the questionnaire.

### 2.5. Statistical analysis

Measurement data are presented as means and standard deviations (SDs), and count data are presented as frequencies and percentages. The baseline data of the groups of participants are summarized as means and SDs (for continuous variables) and numbers and percentages (for categorical variables). Inter-group comparisons were made by performing chi-square tests or one-way difference analyses. The HRs and 95% confidence intervals (CIs) of sleep quality and sleep duration were calculated by establishing a Cox proportional-risk regression model. In addition, a Cox proportional-hazards regression model was established to evaluate the relationship between sleep quality and duration and the incidence rate of arthritis, with good sleep quality and a 5 to 10 hours sleep duration used as the references.

Model 1 was adjusted for age and gender. Model 2 was additionally adjusted for smoking status, alcohol-drinking status, physical activity status, marriage, education level, BMI, and household income. Model 3 was additionally adjusted for place of residence and history of diabetes, heart disease, and stroke. A product term composed of sleep type and lifestyle factors was also added to the models to allow the multiplication and addition interactions between sleep and lifestyle factors to be quantified. In addition, restrictive cubic splines were drawn to explore the nonlinear relationship between participants’ sleep duration and arthritis. Subsequently, a restricted cubic spline regression was conducted: with nodes set at the 5^th^, 35^th^, 65^th^, and 95^th^ percentiles in Model 3, and the median of each exposure variable used as the reference values: to evaluate the continuous change relationship between sleep duration and the incidence rate of arthritis. Moreover, multiple sensitivity analyses were conducted in which all of the above analyses were repeated. Participants who had developed arthritis within 1 year of follow-up were excluded to reduce potential reverse causation bias. In addition, participants with a history of diabetes, stroke, and/or heart disease were excluded because the incidence of arthritis is affected by these major chronic diseases. In addition, participants with abnormal sleep duration or obesity were excluded.

All analyses were conducted using R (version 4.1.1) and Python 3.9. A bilateral *P* value <0.05 was regarded as indicating statistical significance. This study was approved by Ningbo No.2 Hospital.

## 3. Results

### 3.1. Basic information

The average age of the 6844 participants was 85.99 ± 11.14 years, and 54.28% were women. Compared with the participants without arthritis, those with arthritis were significantly different in terms of age (*P* < .001), gender (*P* < .001), alcohol-drinking status (*P* = .031), physical activity status (*P* = .007), marital status (*P* = .033), BMI (*P* < .001), household income (*P* = .003), diabetes (*P* = .02), heart disease (*P* < .001), sleep quality (*P* < .001), and sleep duration (*P* < .001).

### 3.2. Single-factor analysis

Table 1 shows the characteristics of participants stratified by the incidence of arthritis. The majority of the participants lived in rural areas (89.95%), and a minority of the participants lived in urban areas (10.05%). Regarding lifestyle habits, 65.28% of the participants had never smoked, and 67.79% had never consumed alcohol. Regarding physical activity status, 33.58% of the participants engaged in physical activity. In addition, 58.89% of the participants had received no education, and only 10.72% had received higher education. Regarding health status, 3.78% of the participants had diabetes, 11.44% had heart disease, and 7.86% had had a stroke. Regarding sleep quality and duration, 64.26% of the participants had good sleep quality, and 75.03% had a sleep duration of 5 to 10 hours.

**Table 1:** Baseline characteristics for participants from CLHLS sample of China oldest people.

Variable	Level	Overall	Arthritis		*P*
		6844	Yes (799)	No (6045)	
Age (mean [SD])		85.99 (11.14)	82.74 (10.71)	86.42 (11.13)	<.001
Sex (%)	Male	3129 (45.72)	315 (39.42)	2814 (46.55)	<.001
	Female	3715 (54.28)	484 (60.58)	3231 (53.45)	
Residence (%)	Urban	628 (10.05)	74 (9.92)	554 (10.07)	.949
	Rural	5619 (89.95)	672 (90.08)	4947 (89.93)	
Smoking status (%)	Now	1243 (18.32)	140 (17.65)	1103 (18.40)	.077
	Before	1113 (16.40)	110 (13.87)	1003 (16.74)	
	Never	4430 (65.28)	543 (68.47)	3887 (64.86)	
Drinking status (%)	Now	1192 (17.65)	114 (14.54)	1078 (18.06)	.031
	Before	983 (14.56)	109 (13.90)	874 (14.64)	
	Never	4578 (67.79)	561 (71.56)	4017 (67.30)	
Physical activity (%)	Yes	2266 (33.58)	297 (37.88)	1969 (33.01)	.007
	No	4483 (66.42)	487 (62.12)	3996 (66.99)	
Marital status (%)	Married	2443 (35.97)	314 (39.45)	2129 (35.51)	.033
	Other	4349 (64.03)	482 (60.55)	3867 (64.49)	
Education attainment (%)	Never	4016 (58.89)	462 (58.04)	3554 (59.01)	.801
	Low	2072 (30.39)	250 (31.41)	1822 (30.25)	
	High	731 (10.72)	84 (10.55)	647 (10.74)	
BMI (mean [SD])		22.79 (46.71)	20.72 (22.55)	23.08 (49.15)	.233
BMI group (%)	Low	1999 (38.76)	198 (31.23)	1801 (39.82)	<.001
	Normal	2267 (43.96)	299 (47.16)	1968 (43.51)	
	Overweight	618 (11.98)	91 (14.35)	527 (11.65)	
	Obesity	273 (5.29)	46 (7.26)	227 (5.02)	
Income (%)	<4000	1160 (18.57)	149 (20.84)	1011 (18.28)	.003
	4000~10,000	1007 (16.12)	120 (16.78)	887 (16.03)	
	10,000~20,000	1086 (17.38)	90 (12.59)	996 (18.00)	
	≥20,000	2994 (47.93)	356 (49.79)	2638 (47.69)	
Diabetes (%)	Yes	254 (3.78)	42 (5.33)	212 (3.57)	.02
	No	6472 (96.22)	746 (94.67)	5726 (96.43)	
Heart diseases (%)	Yes	772 (11.44)	120 (15.23)	652 (10.94)	<.001
	No	5976 (88.56)	668 (84.77)	5308 (89.06)	
Stroke (%)	Yes	533 (7.86)	50 (6.32)	483 (8.07)	.10
	No	6245 (92.14)	741 (93.68)	5504 (91.93)	
Sleep quality	Good	4398 (64.26)	466 (58.32)	3932 (65.05)	<.001
	General	1660 (24.25)	208 (26.03)	1452 (24.02)	
	Bad	786 (11.48)	125 (15.64)	661 (10.93)	
Sleep time	5–10h	5135 (75.03)	580 (72.59)	4555 (75.35)	<.001
	<5 h	995 (14.54)	166 (20.78)	829 (13.71)	
	>10 h	714 (10.43)	53 (6.63)	661 (10.93)	

BMI = body mass index, CLHLS = Chinese Longitudinal Health and Longevity Survey, SD = standard deviation.

The results of the baseline analysis showed that age, gender, physical activity, marital status, BMI, household income, diabetes, heart disease, sleep quality, and sleep duration were significantly associated with the risk of arthritis (*P* < .05). In the subgroup of participants aged 80 years or under, sleep quality, sleep duration, gender, smoking status, alcohol-drinking status, marital status, education level, and income were significantly associated with the risk of arthritis (*P* < .05). In the subgroup of participants aged over 80 years, only household income, heart disease, and sleep duration were significantly associated with the risk of arthritis (*P* < .05). In the subgroup of middle-aged and older adult male participants, age, household income, sleep quality, and sleep duration were significantly associated with the risk of arthritis. In the subgroup of middle-aged and older adult women participants, age, physical activity status, marital status, education level, BMI, household income, diabetes, heart disease, sleep quality, and sleep duration were significantly associated with the risk of arthritis.

The participants who had never received education, did not engage in physical activity, and had heart disease had a significantly higher incidence rate of arthritis than their counterparts (*P* < .001).

### 3.3. Cox proportional-risk regression model

**Figure 1.**
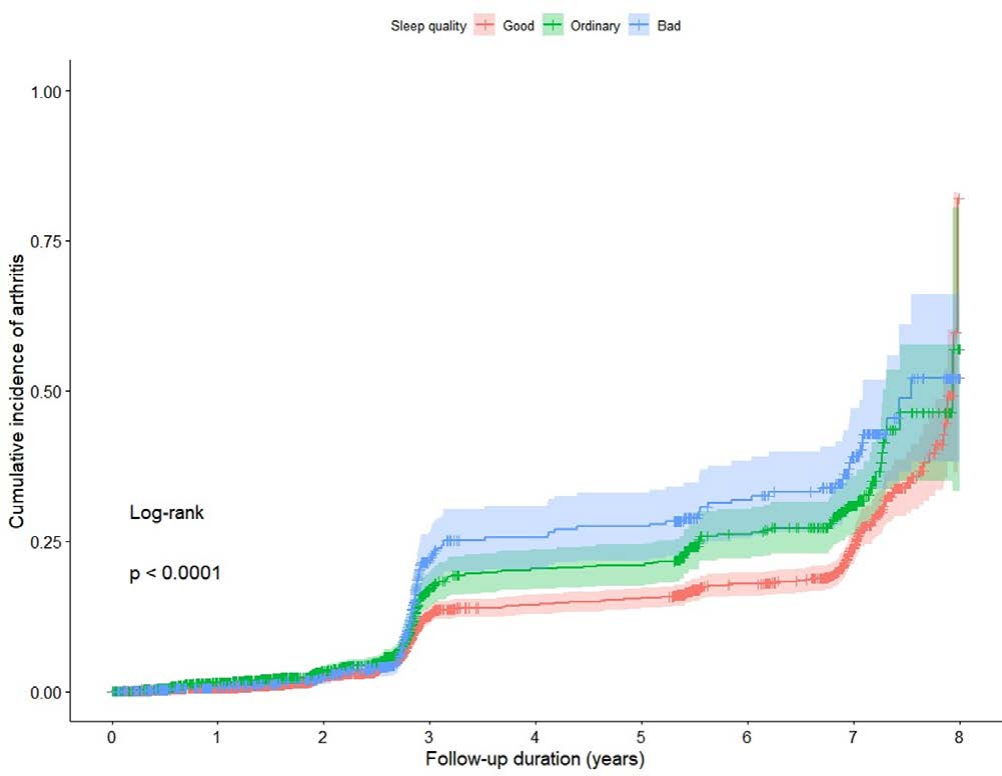
Cumulative incidence of arthritis curves for different sleep qualities.

**Figure 2.**
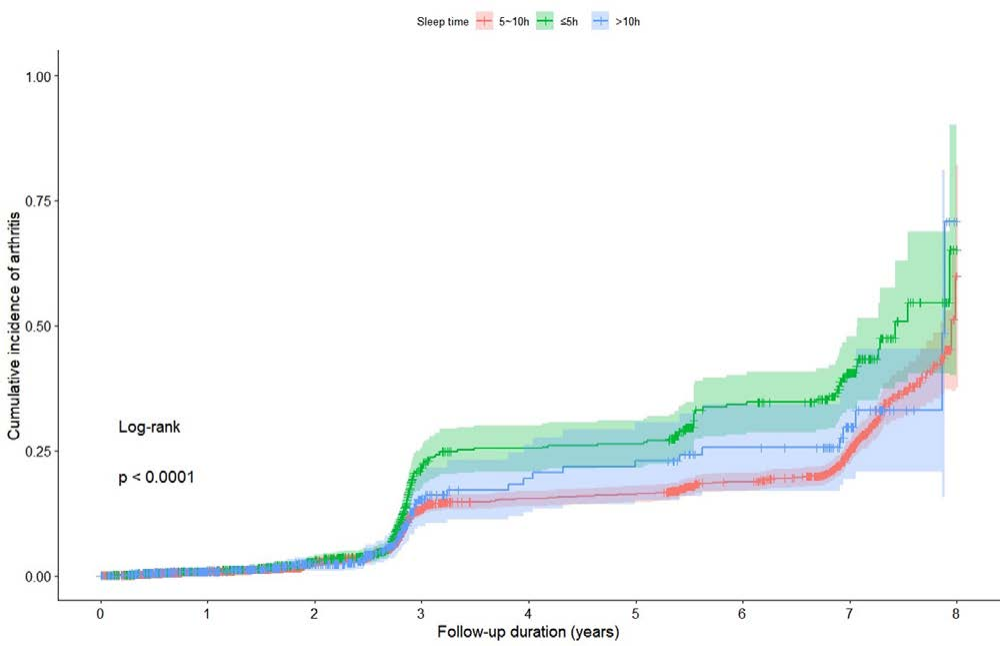
Cumulative incidence of arthritis curves for different sleep time.

After an average follow-up of 3.31 years, 799 (12%) participants had developed arthritis. Figures 1 and 2 show the cumulative estimates of the incidence rate of arthritis based on a Cox proportional-hazards regression model. Compared with participants with poor sleep quality, those with better sleep quality had a significantly lower cumulative incidence rate of arthritis (*P* < .0001 for all log-rank tests; Fig. 1). Moreover, compared with participants who had a sleep duration shorter than 5 hours, those who had a sleep duration of 5 to 10 hours had a significantly lower cumulative incidence rate of arthritis (*P* < .0001 for all log-rank tests; Fig. 2).

The relationship between sleep quality and duration and the incidence rate of arthritis is shown in Table 2. The Cox proportional-risk regression model results show that in the baseline model, participants with poor sleep quality had a 49% higher risk of developing arthritis than those with good sleep quality (HR = 1.49, 95% CI = 1.22–1.82), and that participants with a sleep duration shorter than 5 hours had a 12% higher risk of developing arthritis than those with a sleep duration of 5 to 10 hours (HR = 1.12, 95% CI = 0.84–1.48). After adjustment for age, gender, smoking status, alcohol-drinking status, physical activity status, marital status, education level, BMI, household income, place of residence, and a history of 3 chronic diseases (diabetes, heart disease, and stroke), the incidence rate of arthritis was greater in participants with moderate sleep quality than in those with good sleep quality (HR = 1.24, 95% CI = 1.02–1.52), and greater in participants with poor sleep quality than in those with moderate or good sleep quality (HR = 1.38, 95% CI = 1.09–1.74).

**Table 2:** Crude and adjusted hazards ratio (HR, 95% CI) for arthritis by baseline sleep duration and quality as divided into 3 groups.

Variable		Model 1	Model 2	Model 3
Sleep quality	Good	1 (Reference)	1 (Reference)	1 (Reference)
	Ordinary	1.32 [1.12, 1.55]	1.28 [1.08, 1.5]	1.24 [1.02, 1.52]
	Bad	1.49 [1.22, 1.82]	1.43 [1.17, 1.74]	1.38 [1.09, 1.74]
Sleep time	5–10 h	1 (Reference)	1 (Reference)	1 (Reference)
	<5 h	1.51 [1.27, 1.79]	1.42 [1.19, 1.69]	1.56 [1.27, 1.91]
	>10 h	1.12 [0.84, 1.48]	0.98 [0.74, 1.31]	0.97 [0.7, 1.34]

CI = confidence interval, HR = hazard ratio.

Compared with participants with a sleep duration of 5 to 10 hours, those with a sleep duration shorter than 5 hours had an increased risk of developing arthritis (HR = 1.56, 95% CI = 1.27–1.91), whereas those with a sleep duration longer than 10 hours did not (HR = 0.97, 95% CI = 0.7–1.34; Table 2).

**Figure 3.**
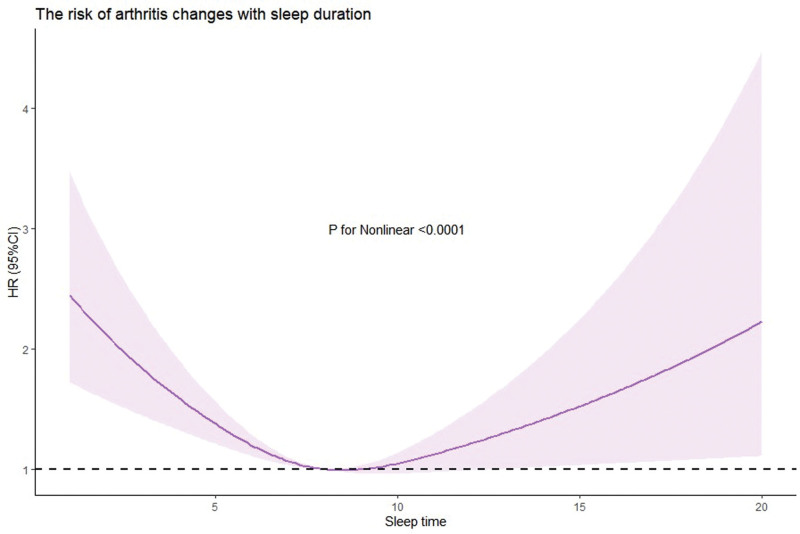
Relationship between sleep duration and risk of developing arthritis.

Analysis of the results of the restricted cubic spline Cox proportional-hazards regression model showed that there was an approximately non-linear dose–response relationship between sleep duration and the incidence rate of arthritis (P nonlinear < 0.0001). Specifically, the risk of developing arthritis significantly decreased as sleep duration increased to 8 hours but slowly increased as sleep duration increased beyond 8 hours (Fig. 3).

A comparison of the differences between each subgroup (those aged ≤ 80 years, aged > 80 years, male, and female subgroups) and the overall population revealed that among participants aged under 80 years, the average age of those with arthritis (73.17 years old) was slightly lower than that of those without arthritis (73.57 years old), whereas among participants aged over 80 years, the average age of those with arthritis (90.83 years old) was substantially lower than that of those without arthritis (92.80 years old). However, this difference was not significant (Supplementary Tables S1–S4, Supplemental Digital Content, http://links.lww.com/MD/N592).

Compared with the overall sample, in participants aged under 80 years, the incidence rate of arthritis (59.56%) was significantly higher in women than in men (40.44%), but in participants aged over 80 years, this difference was not as marked. There was no significant difference in the average BMIs of the various subgroups and the average BMI of the overall sample, but compared with other subgroups, in men and women aged under 80 years, there was a more significant relationship between BMI groups (such as obesity and overweight) and the risk of arthritis. Moreover, in participants aged 80 years and over, poor sleep quality and short sleep duration were significantly associated with the risk of arthritis. Overall, there were significant differences in multiple variables between men and women aged under 80 years and the overall sample.

The Cox proportional-hazards regression model afforded cumulative estimates of the incidence rate of arthritis in each subgroup. This revealed that in the subgroup of participants aged under 80 years, the cumulative incidence rate of arthritis in those with a sleep duration longer than 10 hours was lower than that in those with a sleep duration of 5 to 10 hours (*P* = .0023, *P* < .01; Supplementary Fig. S3[A], Supplemental Digital Content, http://links.lww.com/MD/N591). The results of the other subgroup analyses were largely consistent with the main results for all of the participants (Supplementary Figs. S1–S7, Supplemental Digital Content, http://links.lww.com/MD/N591).

The above-described Cox regression analysis was repeated after controlling for age and gender, and the results of the 2 analyses were compared to afford a complete sensitivity analysis and determine the stability of the results (see Supplementary Table S5, Supplemental Digital Content, http://links.lww.com/MD/N592, where the latter gives sensitivity analysis results for sleep quality, sleep duration, and the incidence rate of arthritis). After adjusting for covariates, it was found that in all subgroups, the participants with moderate sleep quality (HR = 1.15, 95% CI = 0.85–1.56) and those with poor sleep quality (HR = 1.68, 95% CI = 1.22–2.33, *P* < .001) had a greater risk of developing arthritis than those with good sleep quality. In addition, those with a sleep duration shorter than 5 hours had a higher risk of developing arthritis (HR = 1.55, 95% CI = 1.14–2.09) than those with a longer sleep duration. However, the associations observed in all subgroups in participants with a sleep duration longer than 10 hours (HR = 0.99, 95% CI = 0.65–1.50) were not statistically significant. Overall, the sensitivity analysis results were similar to the original analysis results, which confirms the stability of the results.

## 4. Discussion

This prospective cohort study revealed the relationship between sleep quality and duration and the incidence rate of arthritis in a group of older adult Chinese participants during a 7–year follow-up (2011–2018). Specifically, the participants with good sleep quality and moderate sleep duration (5 to 10 hours) had a significantly lower incidence rate of arthritis than those with worse sleep quality and a shorter or longer sleep duration. The implications of our findings are discussed below.

First, this study confirms the relationship between sleep quality and the incidence rate of arthritis. This is consistent with previous studies, in that pain and insomnia are often common symptoms in patients with arthritis.^[10]^ Sleep is affected by pain and may also be affected by chronic inflammation and other mechanisms related to arthritis,^[11]^ such as the immune response, the stress response, and endocrine balance.^[12]^

Second, this study found that there was a relationship between sleep duration and the incidence rate of arthritis, especially in the participants whose sleep duration was too short. Although the percentage increase in the risk of arthritis was relatively small, this finding has significant public health implications, given the ubiquity of sleep problems among older adult people. Moreover, the risk of arthritis was not significantly higher in the participants with a sleep duration that was too long (> 10 hours) than in those with a sleep duration of 5 to 10 hours, which may indicate that in the older adult, it is most important to ensure that sleep duration is not too short.

Third, the analysis of subgroups classified in terms of age and gender revealed that a short sleep duration and poor sleep quality were associated with an increased risk of arthritis in those aged 80 years or under and in those aged over 80 years. However, the risk was greater in the former group than in the latter group. This may be attributable to the former group being more focused on quality of life and health status than the latter group, such that the former group’s sleep status is more susceptible than the latter group’s sleep status to external factors such as life and work pressures, which affect their health status. The analysis of gender subgroups showed that there was a correlation between insufficient sleep and poor sleep quality and an increased risk of arthritis in both genders, but this correlation was more pronounced in women than in men. This may be attributable not only to the unique physiological characteristics and cycles of women but also to the roles and responsibilities typically undertaken by women, which can contribute to stress and sleep disturbances, thereby affecting joint health.

Fourth, certain factors related to health status and lifestyle were found to be associated with an increased risk of arthritis. For example, the participants who had never received an education, did not engage in physical activities, and had heart disease had a significantly higher incidence rate of arthritis than their counterparts. This further highlights the importance of healthy lifestyle interventions to prevent arthritis, especially encouraging older adult people to engage in moderate physical activity.

There are several possible explanations for the above-described correlations. Sleep is crucial for the normal functioning of the immune system. Thus, long periods of insufficient sleep or poor sleep quality may lead to a decrease in immune system function, thereby increasing inflammatory responses,^[13,14]^ which may increase the incidence of arthritis.^[15]^ In addition, prolonged sleep deprivation or poor quality sleep may also increase the body’s stress response, leading to an increase in the secretion of stress hormones such as cortisol,^[16]^ which may exacerbate the symptoms of arthritis. Moreover, sleep is important for maintaining the balance of the endocrine system,^[17]^ and endocrine imbalance may increase the incidence of arthritis. Furthermore, patients with arthritis often experience pain symptoms, which may lead to insomnia. Conversely, insomnia may exacerbate the sensation of pain, forming a vicious cycle. Finally, among participants with the same sleep duration, those who had never received education, did not engage in physical activity, and had heart disease had a significantly higher incidence rate of arthritis than their counterparts.^[18,19]^ These factors may be related to aspects such as lifestyle, dietary habits, and weight control, all of which may affect the risk of arthritis.^[20]^

This study has several limitations. First, the assessment of sleep quality was based on a single self-reported question rather than a standardized and validated toollike the Pittsburgh Sleep Quality Index. Second, the information on arthritis status for deceased participants was obtained from relatives or community doctors and was not always supported by medical reports, which could introduce recall bias. Third, although we controlled for various covariates, residual confounding due to unmeasured factors cannot be ruled out. Future studies should aim to use standardized sleep assessment tools, verify diagnoses with medical records, and explore additional potential confounders.

## 5. Conclusion

This study was the first to examine the relationship between sleep quality and duration and the risk of arthritis among the older adult in China. After controlling for multiple covariates (age; gender; smoking status; alcohol-drinking status; physical activity status; marital status; education level; BMI; household income; place of residence; and history of diabetes, heart disease, and stroke), there was a clear relationship between the quality and duration of sleep and the incidence rate of arthritis in the participants. Those with poor sleep quality and a sleep duration shorter than 5 hours had a higher risk of developing arthritis than those with better sleep quality and a longer sleep duration. Moreover, there was a non-linear association between sleep duration and the incidence of arthritis, especially in subgroups aged over 80 years and those comprising women. These results demonstrate that optimizing sleep quality and duration in the older adult is crucial for reducing their risk of developing arthritis.

## Acknowledgements

We thank all authors for their contributions to the article and confirm that those acknowledged have given permission to be named.

## Author Contributions

**Conceptualization:** HaiTing Wu.

**Data curation:** XinHua Yuan, QingSong Fu.

**Formal analysis:** HaiTing Wu.

**Writing – original draft:** HaiTing Wu, XinHua Yuan, QingSong Fu.

**Writing – review & editing:** HaiTing Wu, XinHua Yuan, QingSong Fu.

## Supplementary Material


