# Supplementary material for: Relationship between sleep quality and duration and the incidence rate of arthritis: A prospective cohort study
Source: Medicine (Baltimore). 2024 Sep 13;103(37):e39641. doi: 10.1097/MD.0000000000039641 (PMC11404932; doi:10.1097/MD.0000000000039641)

Figure S1 Flow chart for inclusion and exclusion of study participants


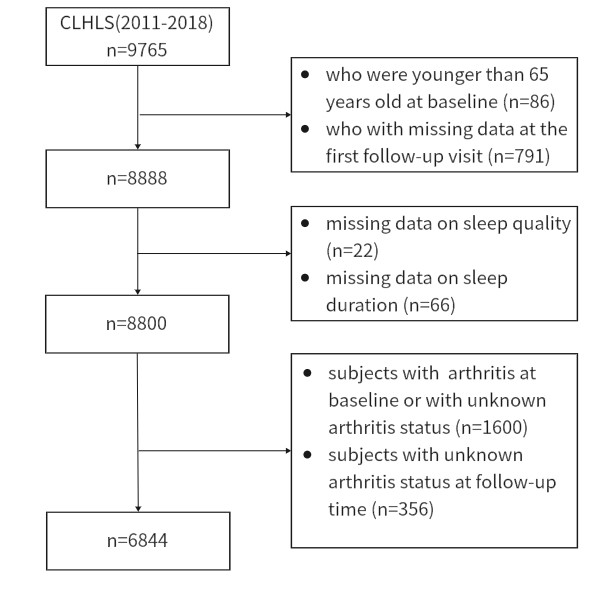


Figure S2 Cumulative incidence of arthritis curves for different sleep quality by age group


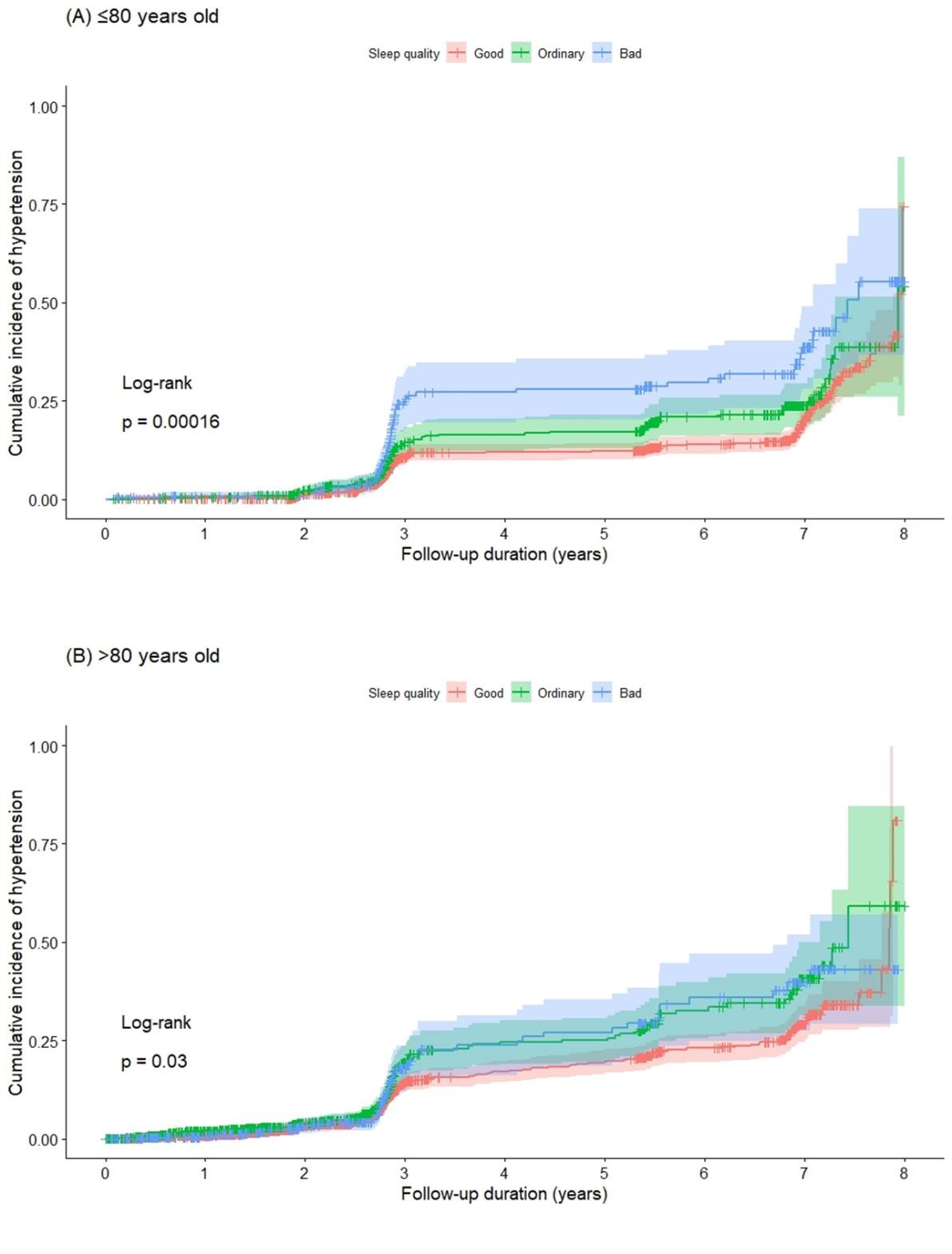


Figure S3 Cumulative incidence of arthritis curves for different sleep quality by sex group


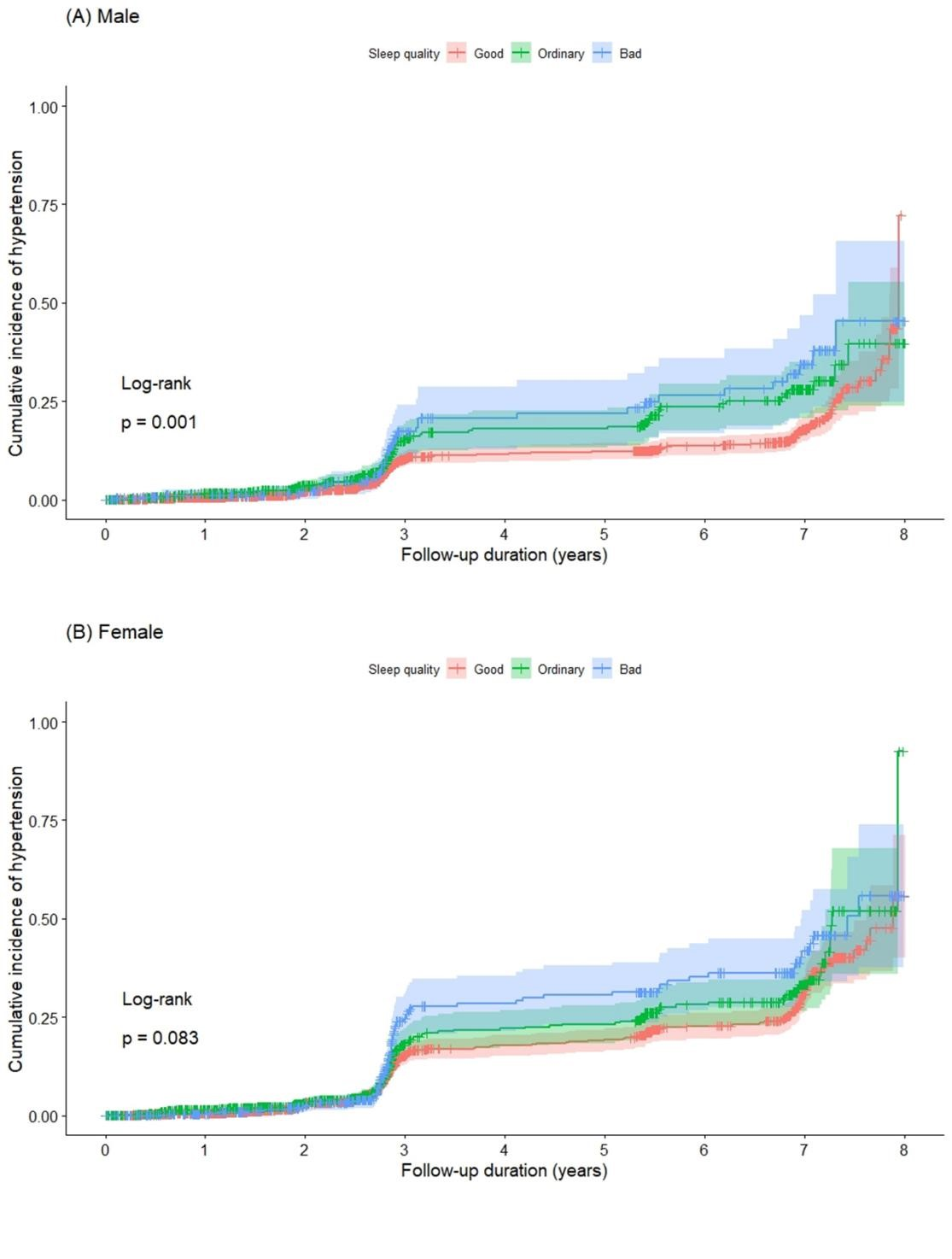


Figure S4 Cumulative incidence of arthritis curves for different sleep time by age group


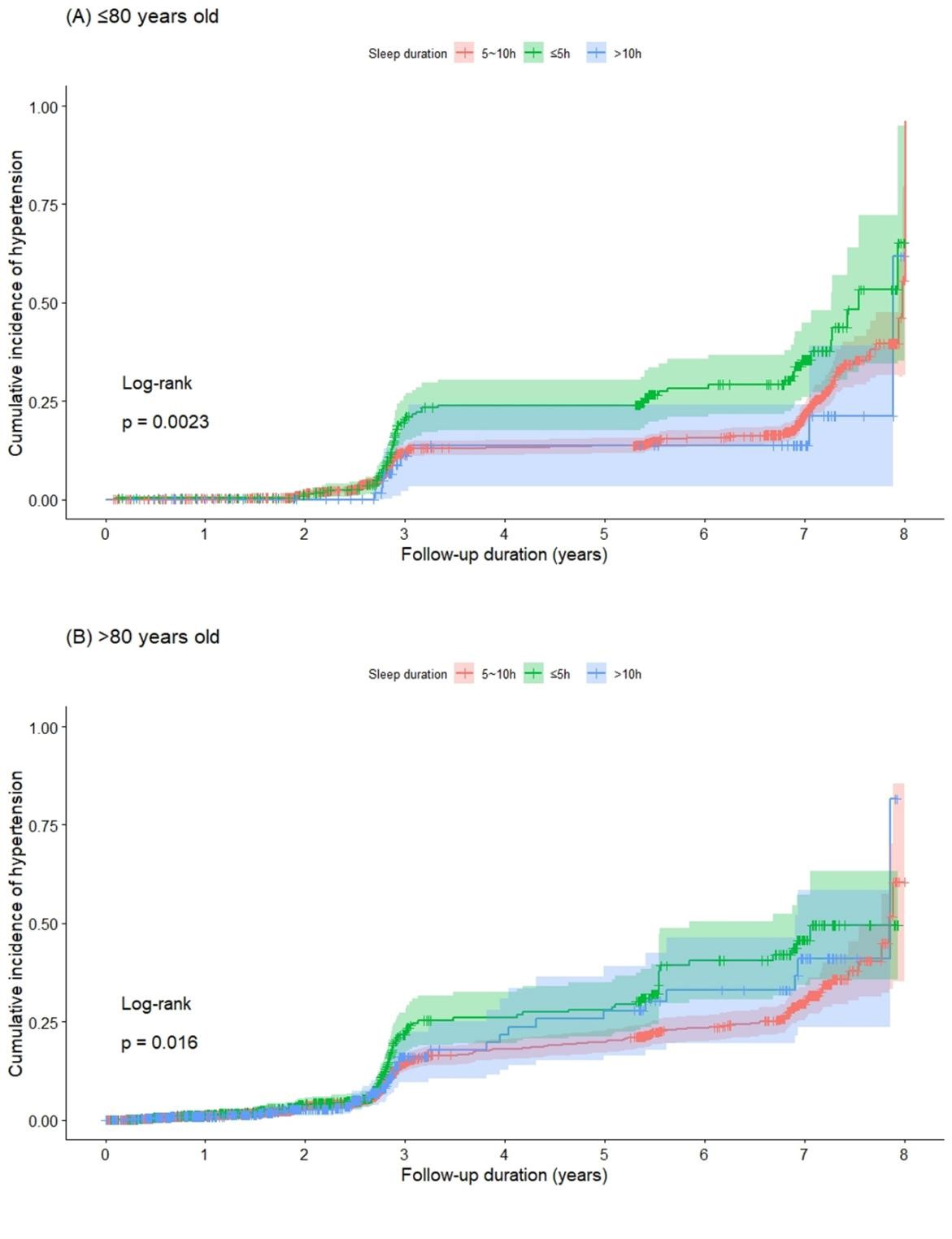


Figure S5 Cumulative incidence of arthritis curves for different sleep quality by sex group


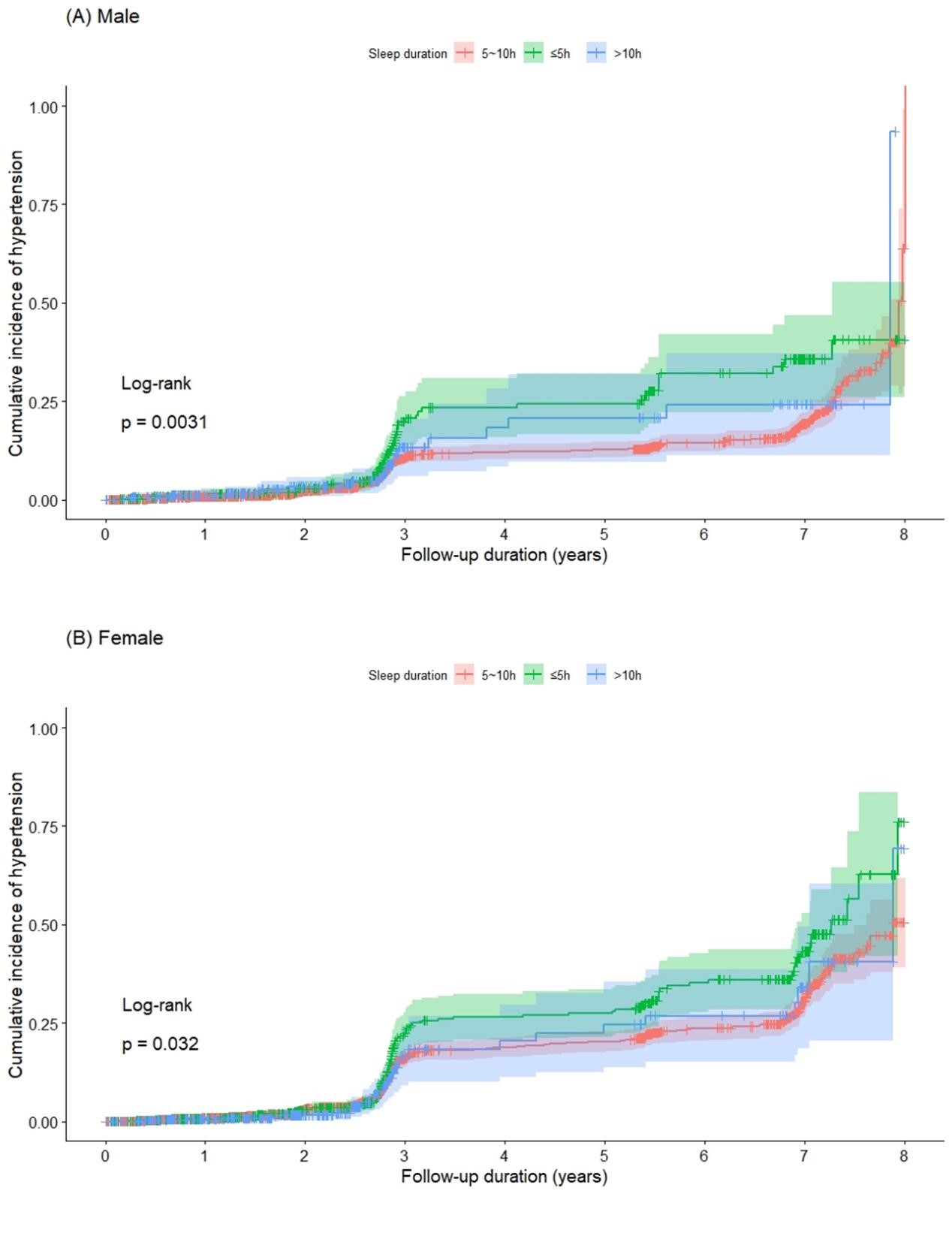


Figure S6 Relationship between sleep duration and risk of developing arthritis by age group


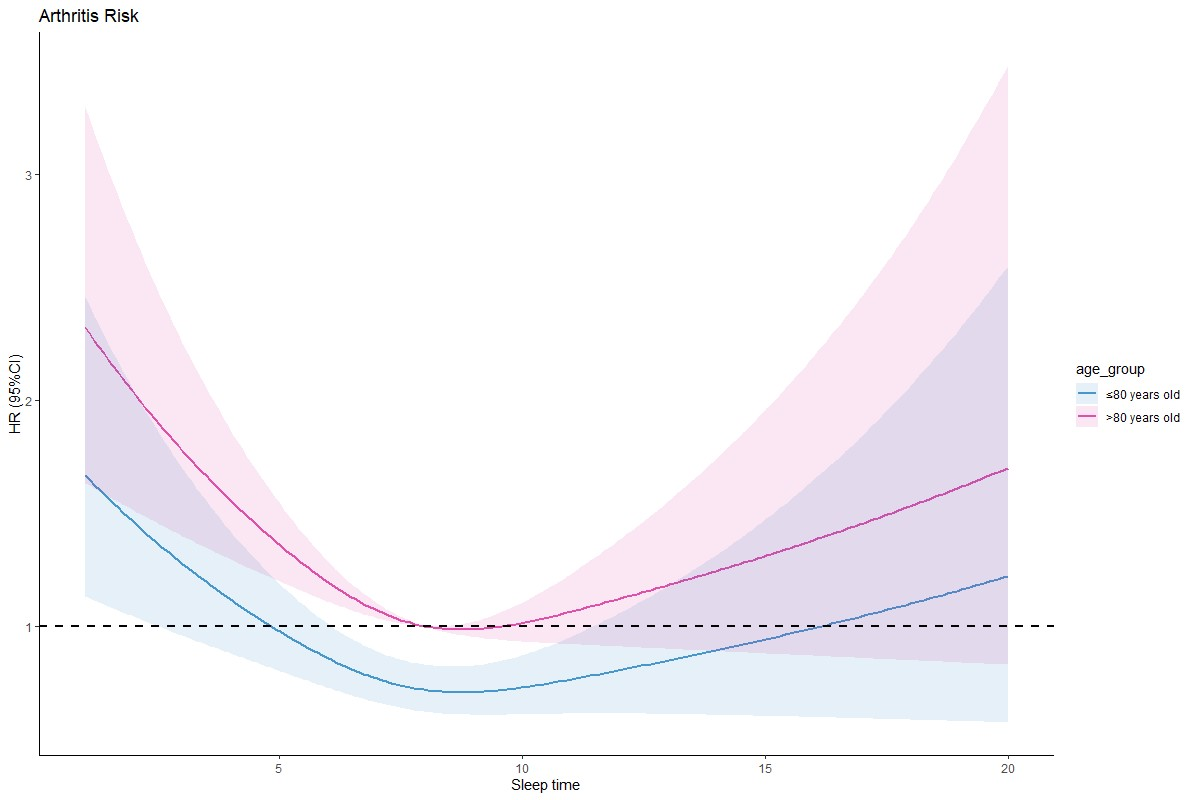


Figure S7 Relationship between sleep duration and risk of developing arthritis by sex group


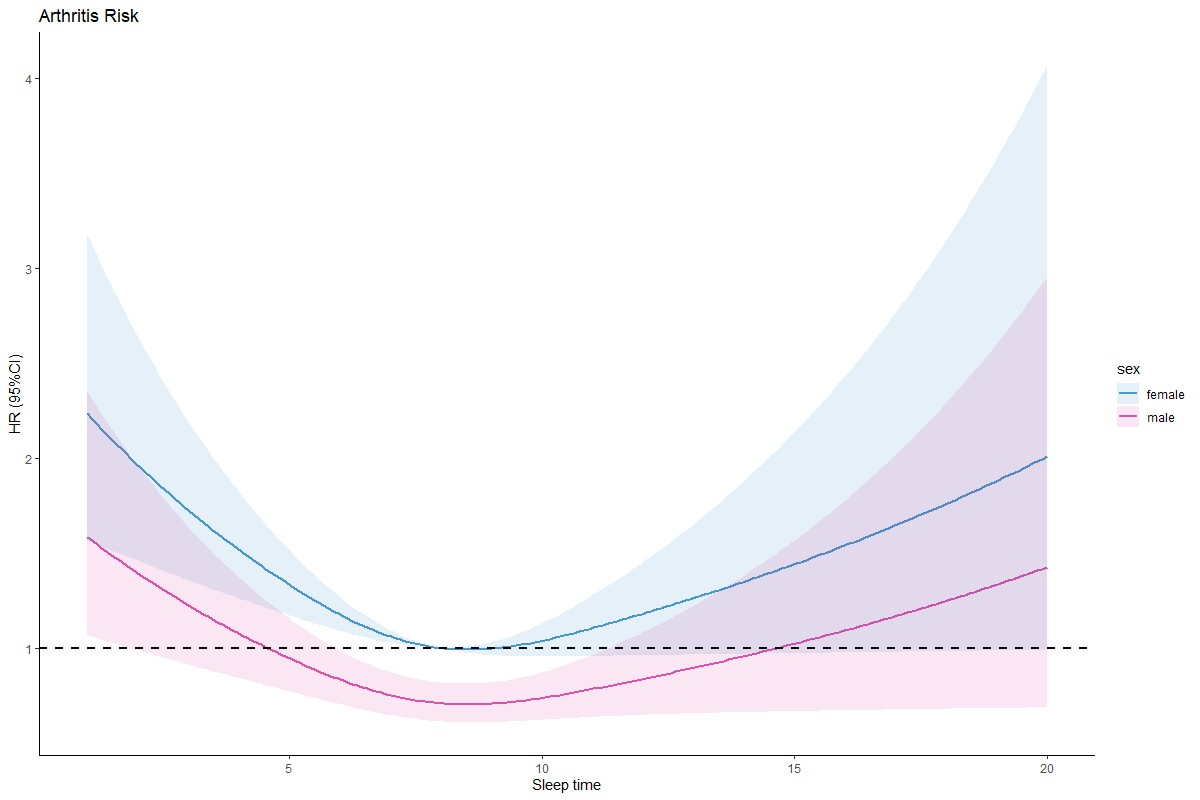

Supplement: Supplementary file 1 [file medi-103-e39641-s001.docx]
