# Supplementary material for: Relationship between sleep quality and duration and the incidence rate of arthritis: A prospective cohort study
Source: Medicine (Baltimore). 2024 Sep 13;103(37):e39641. doi: 10.1097/MD.0000000000039641 (PMC11404932; doi:10.1097/MD.0000000000039641)
Supplement: Supplementary file 2 [file medi-103-e39641-s002.docx]

Table S1 Baseline characteristics for participants aged ≤80 years old from CLHLS sample of China oldest people

|  | level | Overall | Arthritis | | *P* |
| --- | --- | --- | --- | --- | --- |
| Variable |  | 2372 | Yes (366) | No(2006) |  |
| Age (mean (SD)) |  | 73.51 (4.02) | 73.17 (3.92) | 73.57 (4.04) | 0.076 |
| Sex (%) | male | 1307 (55.10) | 148 (40.44) | 1159 (57.78) | <0.001 |
|  | female | 1065 (44.90) | 218 (59.56) | 847 (42.22) |  |
| Residence (%) | urban | 239 (11.24) | 39 (11.34) | 200 (11.22) | 1 |
|  | rural | 1888 (88.76) | 305 (88.66) | 1583 (88.78) |  |
| Smoking status (%) | now | 602 (25.47) | 70 (19.18) | 532 (26.61) | <0.001 |
|  | before | 407 (17.22) | 51 (13.97) | 356 (17.81) |  |
|  | never | 1355 (57.32) | 244 (66.85) | 1111 (55.58) |  |
| Drinking status (%) | now | 526 (22.43) | 57 (15.92) | 469 (23.60) | 0.005 |
|  | before | 330 (14.07) | 51 (14.25) | 279 (14.04) |  |
|  | never | 1489 (63.50) | 250 (69.83) | 1239 (62.36) |  |
| Physical activity (%) | yes | 1028 (43.78) | 168 (46.54) | 860 (43.28) | 0.276 |
|  | no | 1320 (56.22) | 193 (53.46) | 1127 (56.72) |  |
| Marital status (%) | married | 1524 (64.52) | 210 (57.69) | 1314 (65.77) | 0.004 |
|  | other | 838 (35.48) | 154 (42.31) | 684 (34.23) |  |
| Education attainment (%) | never | 927 (39.15) | 163 (44.66) | 764 (38.14) | 0.026 |
|  | low | 995 (42.02) | 148 (40.55) | 847 (42.29) |  |
|  | high | 446 (18.83) | 54 (14.79) | 392 (19.57) |  |
| BMI (mean (SD)) |  | 22.36 (47.41) | 21.93 (19.50) | 22.44 (51.19) | 0.863 |
| BMI group (%) | low | 405 (22.63) | 51 (17.17) | 354 (23.71) | 0.018 |
|  | normal | 928 (51.84) | 154 (51.85) | 774 (51.84) |  |
|  | overweight | 337 (18.83) | 64 (21.55) | 273 (18.29) |  |
|  | obesity | 120 (6.70) | 28 (9.43) | 92 (6.16) |  |
| Income (%) | <4000 | 398 (18.25) | 73 (21.99) | 325 (17.58) | 0.047 |
|  | 4000~10000 | 370 (16.96) | 50 (15.06) | 320 (17.31) |  |
|  | 10000~20000 | 399 (18.29) | 47 (14.16) | 352 (19.04) |  |
|  | ≥20000 | 1014 (46.49) | 162 (48.80) | 852 (46.08) |  |
| Diabetes (%) | Yes | 155 (6.62) | 30 (8.33) | 125 (6.30) | 0.19 |
|  | No | 2188 (93.38) | 330 (91.67) | 1858 (93.70) |  |
| Heart diseases (%) | Yes | 305 (13.01) | 58 (16.11) | 247 (12.45) | 0.07 |
|  | No | 2039 (86.99) | 302 (83.89) | 1737 (87.55) |  |
| Stroke (%) | Yes | 215 (9.13) | 27 (7.42) | 188 (9.44) | 0.257 |
|  | No | 2140 (90.87) | 337 (92.58) | 1803 (90.56) |  |
| Sleep quality | Good | 1556 (65.60) | 213 (58.20) | 1343 (66.95) | <0.001 |
|  | general | 540 (22.77) | 87 (23.77) | 453 (22.58) |  |
|  | bad | 276 (11.64) | 66 (18.03) | 210 (10.47) |  |
| Sleep time | 5-10h | 1946 (82.04) | 284 (77.60) | 1662 (82.85) | 0.002 |
|  | <5h | 337 (14.21) | 73 (19.95) | 264 (13.16) |  |
|  | >10h | 89 (3.75) | 9 (2.46) | 80 (3.99) |  |

Table S2 Baseline characteristics for participants aged >80 years old from CLHLS sample of China oldest people

|  | level | Overall | | Arthritis | | *P* |
| --- | --- | --- | --- | --- | --- | --- |
| Variable |  | 4472 | | Yes (433) | No (4039) |  |
| Age (mean (SD)) |  | 92.61 (7.41) | 90.83 (7.47) | | 92.80 (7.38) | <0.001 |
| Sex (%) | male | 1822 (40.74) | 167 (38.57) | | 1655 (40.98) | 0.359 |
|  | female | 2650 (59.26) | 266 (61.43) | | 2384 (59.02) |  |
| Residence (%) | urban | 389 (9.44) | 35 (8.71) | | 354 (9.52) | 0.659 |
|  | rural | 3731 (90.56) | 367 (91.29) | | 3364 (90.48) |  |
| Smoking status (%) | now | 641 (14.50) | 70 (16.36) | | 571 (14.30) | 0.28 |
|  | before | 706 (15.97) | 59 (13.79) | | 647 (16.20) |  |
|  | never | 3075 (69.54) | 299 (69.86) | | 2776 (69.50) |  |
| Drinking status (%) | now | 666 (15.11) | 57 (13.38) | | 609 (15.29) | 0.374 |
|  | before | 653 (14.81) | 58 (13.62) | | 595 (14.94) |  |
|  | never | 3089 (70.08) | 311 (73.00) | | 2778 (69.76) |  |
| Physical activity (%) | yes | 1238 (28.13) | 129 (30.50) | | 1109 (27.88) | 0.279 |
|  | no | 3163 (71.87) | 294 (69.50) | | 2869 (72.12) |  |
| Marital status (%) | married | 919 (20.74) | 104 (24.07) | | 815 (20.39) | 0.083 |
|  | other | 3511 (79.26) | 328 (75.93) | | 3183 (79.61) |  |
| Education attainment (%) | never | 3089 (69.40) | 299 (69.37) | | 2790 (69.40) | 0.866 |
|  | low | 1077 (24.20) | 102 (23.67) | | 975 (24.25) |  |
|  | high | 285 (6.40) | 30 (6.96) | | 255 (6.34) |  |
| BMI (mean (SD)) |  | 23.01 (46.34) | 19.65 (24.91) | | 23.39 (48.12) | 0.16 |
| BMI group (%) | low | 1594 (47.34) | 147 (43.62) | | 1447 (47.76) | 0.446 |
|  | normal | 1339 (39.77) | 145 (43.03) | | 1194 (39.41) |  |
|  | overweight | 281 (8.35) | 27 (8.01) | | 254 (8.38) |  |
|  | obesity | 153 (4.54) | 18 (5.34) | | 135 (4.46) |  |
| Income (%) | <4000 | 762 (18.74) | 76 (19.84) | | 686 (18.63) | 0.015 |
|  | 4000~10000 | 637 (15.67) | 70 (18.28) | | 567 (15.40) |  |
|  | 10000~20000 | 687 (16.90) | 43 (11.23) | | 644 (17.49) |  |
|  | ≥20000 | 1980 (48.70) | 194 (50.65) | | 1786 (48.49) |  |
| Diabetes (%) | Yes | 99 (2.26) | 12 (2.80) | | 87 (2.20) | 0.53 |
|  | No | 4284 (97.74) | 416 (97.20) | | 3868 (97.80) |  |
| Heart diseases (%) | Yes | 467 (10.60) | 62 (14.49) | | 405 (10.19) | 0.008 |
|  | No | 3937 (89.40) | 366 (85.51) | | 3571 (89.81) |  |
| Stroke (%) | Yes | 318 (7.19) | 23 (5.39) | | 295 (7.38) | 0.156 |
|  | No | 4105 (92.81) | 404 (94.61) | | 3701 (92.62) |  |
| Sleep quality | Good | 2842 (63.55) | 253 (58.43) | | 2589 (64.10) | 0.059 |
|  | general | 1120 (25.04) | 121 (27.94) | | 999 (24.73) |  |
|  | bad | 510 (11.40) | 59 (13.63) | | 451 (11.17) |  |
| Sleep time | 5-10h | 3189 (71.31) | 296 (68.36) | | 2893 (71.63) | <0.001 |
|  | <5h | 658 (14.71) | 93 (21.48) | | 565 (13.99) |  |
|  | >10h | 625 (13.98) | 44 (10.16) | | 581 (14.38) |  |

Table S3 Baseline characteristics for male from CLHLS sample of China oldest people

|  | level | Overall | Arthritis | | *P* |
| --- | --- | --- | --- | --- | --- |
| Variable |  | 3129 | Yes (315) | No (2814) |  |
| Age (mean (SD)) |  | 83.35 (10.14) | 81.60 (9.54) | 83.55 (10.19) | 0.001 |
| Residence (%) | urban | 311 (10.92) | 34 (11.49) | 277 (10.85) | 0.815 |
|  | rural | 2538 (89.08) | 262 (88.51) | 2276 (89.15) |  |
| Smoking status (%) | now | 1033 (33.26) | 118 (37.46) | 915 (32.78) | 0.247 |
|  | before | 901 (29.01) | 85 (26.98) | 816 (29.24) |  |
|  | never | 1172 (37.73) | 112 (35.56) | 1060 (37.98) |  |
| Drinking status (%) | now | 889 (28.84) | 87 (28.34) | 802 (28.90) | 0.729 |
|  | before | 727 (23.59) | 78 (25.41) | 649 (23.39) |  |
|  | never | 1466 (47.57) | 142 (46.25) | 1324 (47.71) |  |
| Physical activity (%) | yes | 1212 (39.19) | 126 (40.65) | 1086 (39.02) | 0.621 |
|  | no | 1881 (60.81) | 184 (59.35) | 1697 (60.98) |  |
| Marital status (%) | married | 1685 (54.28) | 186 (59.24) | 1499 (53.73) | 0.072 |
|  | other | 1419 (45.72) | 128 (40.76) | 1291 (46.27) |  |
| Education attainment (%) | never | 1030 (33.02) | 106 (33.76) | 924 (32.94) | 0.958 |
|  | low | 1508 (48.35) | 150 (47.77) | 1358 (48.41) |  |
|  | high | 581 (18.63) | 58 (18.47) | 523 (18.65) |  |
| BMI (mean (SD)) |  | 23.07 (35.42) | 22.08 (25.25) | 23.18 (36.41) | 0.642 |
| BMI group(%) | low | 746 (31.24) | 65 (26.21) | 681 (31.82) | 0.107 |
|  | normal | 1220 (51.09) | 128 (51.61) | 1092 (51.03) |  |
|  | overweight | 309 (12.94) | 38 (15.32) | 271 (12.66) |  |
|  | obesity | 113 (4.73) | 17 (6.85) | 96 (4.49) |  |
| Income (%) | <4000 | 518 (17.97) | 56 (19.58) | 462 (17.79) | 0.009 |
|  | 4000~10000 | 452 (15.68) | 62 (21.68) | 390 (15.02) |  |
|  | 10000~20000 | 484 (16.79) | 37 (12.94) | 447 (17.21) |  |
|  | ≥20000 | 1429 (49.57) | 131 (45.80) | 1298 (49.98) |  |
| Diabetes (%) | Yes | 112 (3.63) | 11 (3.54) | 101 (3.64) | 1.00 |
|  | No | 2972 (96.37) | 300 (96.46) | 2672 (96.36) |  |
| Heart diseases (%) | Yes | 340 (11.00) | 41 (13.27) | 299 (10.75) | 0.213 |
|  | No | 2750 (89.00) | 268 (86.73) | 2482 (89.25) |  |
| Stroke (%) | Yes | 273 (8.79) | 22 (7.07) | 251 (8.98) | 0.307 |
|  | No | 2832 (91.21) | 289 (92.93) | 2543 (91.02) |  |
| Sleep quality | Good | 2169 (69.32) | 196 (62.22) | 1973 (70.11) | 0.009 |
|  | general | 682 (21.80) | 80 (25.40) | 602 (21.39) |  |
|  | bad | 278 (8.88) | 39 (12.38) | 239 (8.49) |  |
| Sleep time | 5-10h | 2483 (79.35) | 241 (76.51) | 2242 (79.67) | 0.004 |
|  | <5h | 361 (11.54) | 53 (16.83) | 308 (10.95) |  |
|  | >10h | 285 (9.11) | 21 (6.67) | 264 (9.38) |  |

Table S4 Baseline characteristics for female from CLHLS sample of China oldest people

|  | level | Overall | Arthritis | | *P* |
| --- | --- | --- | --- | --- | --- |
| Variable |  | 3715 | Yes (484) | No (3231) |  |
| Age (mean (SD)) |  | 88.21 (11.46) | 83.48 (11.36) | 88.92 (11.30) | <0.001 |
| Residence (%) | urban | 317 (9.33) | 40 (8.89) | 277 (9.40) | 0.797 |
|  | rural | 3081 (90.67) | 410 (91.11) | 2671 (90.60) |  |
| Smoking status (%) | now | 210 (5.71) | 22 (4.60) | 188 (5.87) | 0.448 |
|  | before | 212 (5.76) | 25 (5.23) | 187 (5.84) |  |
|  | never | 3258 (88.53) | 431 (90.17) | 2827 (88.29) |  |
| Drinking status (%) | now | 303 (8.25) | 27 (5.66) | 276 (8.64) | 0.072 |
|  | before | 256 (6.97) | 31 (6.50) | 225 (7.04) |  |
|  | never | 3112 (84.77) | 419 (87.84) | 2693 (84.31) |  |
| Physical activity (%) | yes | 1054 (28.83) | 171 (36.08) | 883 (27.75) | <0.001 |
|  | no | 2602 (71.17) | 303 (63.92) | 2299 (72.25) |  |
| Marital status (%) | married | 758 (20.55) | 128 (26.56) | 630 (19.65) | 0.001 |
|  | other | 2930 (79.45) | 354 (73.44) | 2576 (80.35) |  |
| Education attainment (%) | never | 2986 (80.70) | 356 (73.86) | 2630 (81.73) | <0.001 |
|  | low | 564 (15.24) | 100 (20.75) | 464 (14.42) |  |
|  | high | 150 (4.05) | 26 (5.39) | 124 (3.85) |  |
| BMI (mean (SD)) |  | 22.54 (54.61) | 19.84 (20.61) | 22.98 (58.27) | 0.295 |
| BMI group (%) | low | 1253 (45.25) | 133 (34.46) | 1120 (47.00) | <0.001 |
|  | normal | 1047 (37.81) | 171 (44.30) | 876 (36.76) |  |
|  | overweight | 309 (11.16) | 53 (13.73) | 256 (10.74) |  |
|  | obesity | 160 (5.78) | 29 (7.51) | 131 (5.50) |  |
| Income (%) | <4000 | 642 (19.08) | 93 (21.68) | 549 (18.71) | 0.001 |
|  | 4000~10000 | 555 (16.50) | 58 (13.52) | 497 (16.93) |  |
|  | 10000~20000 | 602 (17.90) | 53 (12.35) | 549 (18.71) |  |
|  | ≥20000 | 1565 (46.52) | 225 (52.45) | 1340 (45.66) |  |
| Diabetes (%) | Yes | 142 (3.90) | 31 (6.50) | 111 (3.51) | 0.003 |
|  | No | 3500 (96.10) | 446 (93.50) | 3054 (96.49) |  |
| Heart diseases (%) | Yes | 432 (11.81) | 79 (16.49) | 353 (11.10) | 0.001 |
|  | No | 3226 (88.19) | 400 (83.51) | 2826 (88.90) |  |
| Stroke (%) | Yes | 260 (7.08) | 28 (5.83) | 232 (7.27) | 0.296 |
|  | No | 3413 (92.92) | 452 (94.17) | 2961 (92.73) |  |
| Sleep quality | Good | 2229 (60.00) | 270 (55.79) | 1959 (60.63) | 0.014 |
|  | general | 978 (26.33) | 128 (26.45) | 850 (26.31) |  |
|  | bad | 508 (13.67) | 86 (17.77) | 422 (13.06) |  |
| Sleep time | 5-10h | 2652 (71.39) | 339 (70.04) | 2313 (71.59) | <0.001 |
|  | <5h | 634 (17.07) | 113 (23.35) | 521 (16.13) |  |
|  | >10h | 429 (11.55) | 32 (6.61) | 397 (12.29) |  |

Table S5 Associations of sleep quality and sleep time with incidence of arthritis by age and sex

|  |  | |  | | Model 1 | Model 2 | Model 3 |
| --- | --- | --- | --- | --- | --- | --- | --- |
| ≤80 years old | | sleep quality | |  | 1(Reference) | 1(Reference) | 1(Reference) |
|  | |  | |  | 1.29[1,1.65] | 1.22[0.95,1.57] | 1.15[0.85,1.56] |
|  | |  | |  | 1.77[1.34,2.34] | 1.63[1.23,2.15] | 1.68[1.22,2.33] |
|  | | sleep time | |  | 1(Reference) | 1(Reference) | 1(Reference) |
|  | |  | |  | 1.54[1.19,2] | 1.42[1.1,1.85] | 1.55[1.14,2.09] |
|  | |  | |  | 0.77[0.4,1.5] | 0.74[0.38,1.44] | 0.74[0.34,1.58] |
| > 80 years old | | sleep quality | |  | 1(Reference) | 1(Reference) | 1(Reference) |
|  | |  | |  | 1.32[1.06,1.64] | 1.3[1.05,1.62] | 1.34[1.03,1.74] |
|  | |  | |  | 1.26[0.95,1.68] | 1.24[0.93,1.64] | 1.08[0.76,1.52] |
|  | | sleep time | |  | 1(Reference) | 1(Reference) | 1(Reference) |
|  | |  | |  | 1.42[1.13,1.8] | 1.4[1.11,1.77] | 1.49[1.13,1.97] |
|  | |  | |  | 1.1[0.8,1.51] | 1.1[0.8,1.5] | 1.08[0.75,1.55] |
| Male | | sleep quality | |  | 1(Reference) | 1(Reference) | 1(Reference) |
|  | |  | |  | 1.48[1.14,1.92] | 1.45[1.12,1.88] | 1.23[0.89,1.7] |
|  | |  | |  | 1.64[1.16,2.32] | 1.63[1.15,2.29] | 1.34[0.88,2.04] |
|  | | sleep time | |  | 1(Reference) | 1(Reference) | 1(Reference) |
|  | |  | |  | 1.65[1.23,2.23] | 1.6[1.19,2.15] | 1.58[1.09,2.28] |
|  | |  | |  | 1.25[0.8,1.96] | 1.1[0.7,1.72] | 0.97[0.57,1.65] |
| Female | | sleep quality | |  | 1(Reference) | 1(Reference) | 1(Reference) |
|  | |  | |  | 1.15[0.93,1.42] | 1.15[0.93,1.42] | 1.22[0.95,1.57] |
|  | |  | |  | 1.31[1.03,1.68] | 1.32[1.03,1.68] | 1.35[1.02,1.8] |
|  | | sleep time | |  | 1(Reference) | 1(Reference) | 1(Reference) |
|  | |  | |  | 1.34[1.08,1.66] | 1.33[1.08,1.65] | 1.56[1.22,1.99] |
|  | |  | |  | 1.01[0.7,1.45] | 0.97[0.67,1.39] | 0.99[0.65,1.5] |
